# Thiol-reactive reagents inhibits intracellular trafficking of human papillomavirus type 16 pseudovirions by binding to cysteine residues of major capsid protein L1

**DOI:** 10.1186/1743-422X-4-110

**Published:** 2007-10-26

**Authors:** Yoshiyuki Ishii, Kazunari Kondo, Tamae Matsumoto, Keiko Tanaka, Fumiko Shinkai-Ouchi, Ken'ichi Hagiwara, Tadahito Kanda

**Affiliations:** 1 Center for Pathogen Genomics, National Institute of Infectious Diseases, 1-23-1 Toyama, Shinjuku-ku, Tokyo 162-8640, Japan; 2 Department of Pathology, National Institute of Infectious Diseases, 1-23-1 Toyama, Shinjuku-ku, Tokyo 162-8640, Japan; 3 Department of Biochemistry and Cell Biology, National Institute of Infectious Diseases, 1-23-1 Toyama, Shinjuku-ku, Tokyo 162-8640, Japan

## Abstract

**Background:**

A human papillomavirus (HPV) virion is composed of capsid proteins L1 and L2. Several cysteine residues are located on L1 of various HPVs at markedly similar relative positions, suggesting their important functions. Although the authentic virions cannot be studied with cultured cells, surrogate pseudovirions consisting of capsid and reporter plasmid are available for studies dealing with infectivity.

**Results:**

HPV type16-pseudovirions (16PVs) were found to lose their infectivity after incubation with thiol-reactive reagents [biotin polyethyleneoxide iodoacetamide (BPEOIA), 5,5'-dithiobis(2-nitrobenzoic acid) (DTNB), N-ethylmaleimide (NEM), 4-(N-maleimido)benzyl-trimethylammonium iodide (MBTA), and [2-(trimethylammonium)ethyl] methanethiosulfonate bromide (MTSET)]. A labelled streptavidin was detected to bind to the complex of BPEOIA and L1 of the 16PVs incubated with BPEOIA. The analysis of molecular mass of trypsin-fragments derived from the complex of the BPEOIA and L1 indicated that BPEOIA bound to at least C146, C225, and C229. No appreciable change of the 16PVs carrying DTNB or NEM was detected by sedimentation analysis or electron microscopy. The 16PVs carrying DTNB or NEM were able to bind to and enter HeLa cells but degraded before they reached the perinuclear region.

**Conclusion:**

HPV16 L1 C146, C225, and C229 have free thiol, which are accessible to BPEOIA, DTNB, NEM, MBTA, and MTSET. Binding of DTNB or NEM to the thiols may cause conformational changes that result in the inhibition of the entry and trafficking of the 16PVs.

## Background

Human papillomavirus (HPV) is a non-enveloped icosahedral particle (55 nm in diameter) containing an 8-kb double-strand circular DNA [1]. An HPV-capsid is composed of 360 molecules of major capsid protein L1 and 12 molecules of minor capsid protein L2 [2]. To date more than 100 HPV genotypes, which are classified by DNA homology, have been cloned and are grouped into mucosal and cutaneous types from the tissue tropism [3]. Among mucosal types 15 HPVs detected in cervical cancer, the second most frequent gynaecological malignancy in the world, are called as high-risk types and those detected in benign lesions, such as condyloma, are called as low-risk types [4]. HPV type 16 (HPV16) is believed to account for 50% of cervical cancer [4].

HPVs infect basal cells of the epithelium through microlesions and replicate only in the differentiating cells [5]. These cells are difficult to culture in vitro; hence, no tissue culture system for the large-scale propagation of HPVs is available at present. By using surrogate systems the expression of L1 and L2 in cells harboring episomal copies of expression plasmid results in packaging of the episomal DNA into the HPV capsids to produce infectious pseudovirions (PVs)[6,7]. These PVs are used as a surrogate virus to analyse early steps of HPV infection to cells and to detect neutralizing activity of anti-HPV antibodies [8-13].

**Figure 1:**
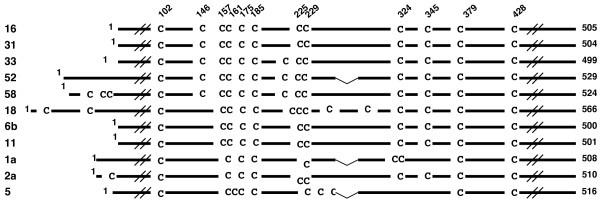
**Alignment of L1 amino acid sequences of papillomaviruses**. Numbers to the left represent human papillomavirus types. Numbers on the top represent amino acid numbers for cysteines in HPV 16 L1 (positions in L1), starting from the N-terminus. The number of total amino acids constituting each L1 is shown to the right.

An L1 molecule of various HPVs contains several cysteine residues at markedly similar relative positions (Fig. 1), strongly suggesting that these cysteine residues play important roles in the structure and the function of the HPV capsids. Previous studies have shown that cysteine residue at amino acid (aa) 175 (C175) and C428 in HPV16 L1 (505 amino acids long) are involved in the intermolecular disulfide bonding that contributes to the assembly of the capsid [14]. The functions of the other L1 cysteine residues are not known.

In this study we attempted to know whether thiol-reactive reagents affect infectivity of HPV16 PVs (16Pvs) by binding to the L1 cysteine residues.

## Results

### Infectivity of the 16PVs that have bound to thiol-reactive reagents

**Figure 2:**
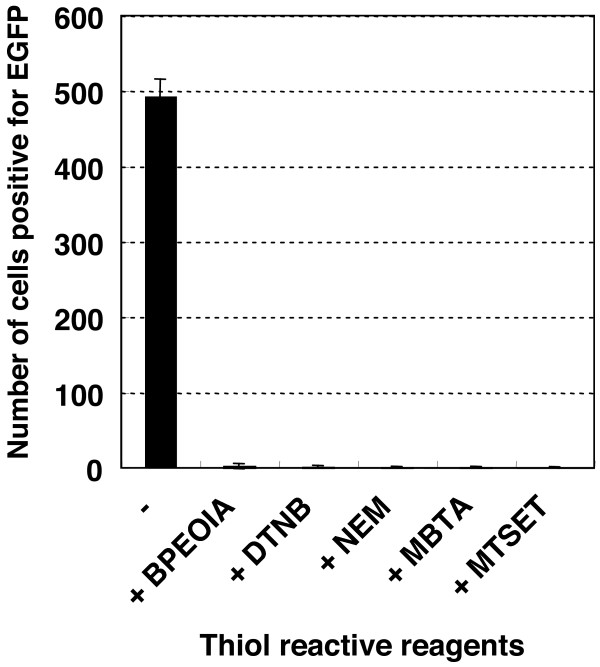
**Infectivity of the 16PVs that have bound to BPEOIA, NEM, DTNB, MBTA, or MTSET**. The 16PVs were incubated with the thiol-reactive reagent indicated at 37°C for 2 h. The samples were diluted by 1000-fold and added to HeLa cells. The cells were incubated for 2 days and harvested. The cells expressing EGFP were counted by a FACS. BPEOIA: biotin polyethyleneoxide iodoacetamid, NEM: N-ethylmaleimide, DTNB: 5,5'-dithiobis(2-nitrobenzoic acid), MBTA: 4-(N-maleimido)benzyl-trimethylammonium iodide, MTSET: [2-(trimethylammonium)ethyl] methanethiosulfonate bromide.

The 16PVs was found to lose their infectivity for HeLa cells after binding to thiol-reactive reagents: biotin polyethyleneoxide iodoacetamide (BPEOIA), 5,5'-dithiobis(2-nitrobenzoic acid) (DTNB), N-ethylmaleimide (NEM), 4-(N-maleimido)benzyl-trimethylammonium iodide (MBTA), and [2-(trimethylammonium)ethyl] methanethiosulfonate bromide (MTSET). 16PVs were incubated with BPEOIA (1 mM), DTNB (2 mM), NEM (2 mM), MBTA (2 mM), or MTSET (2 mM) for 2 h at 37°C. After dilution at 1 to 1,000 the 16PVs were inoculated to the cells. The number of the infected cells, which expressed EGFP, was counted 2 days later. The HeLa cells inoculated with the 16PVs incubated with these thiol-reactive reagents did not express EGFP (Fig. 2). Like HeLa cells, SiHa and 293TT cells inoculated with the 16PVs that had incubated with DTNB did not express EGFP (data not presented). The data indicate that these thiol-reactive reagents inhibited infectivity of the 16PVs.

DTNB did not affect the cellular susceptibility to 16PVs. The normal 16PVs infected HeLa cells that had been cultured in the growth medium containing DTNB (2 mM) for 2 h at 37°C and washed once with fresh growth medium (data not presented). Furthermore HeLa cells cultured with growth medium containing 2 mM of DTNB, MBTA or MTSET for 2 days grew and were maintained normally, strongly suggesting the reagents were not harmful to HeLa cells at the concentration of 2 mM.

### Binding of BPEOIA to the L1 cysteine residues of 16PV

**Figure 3:**
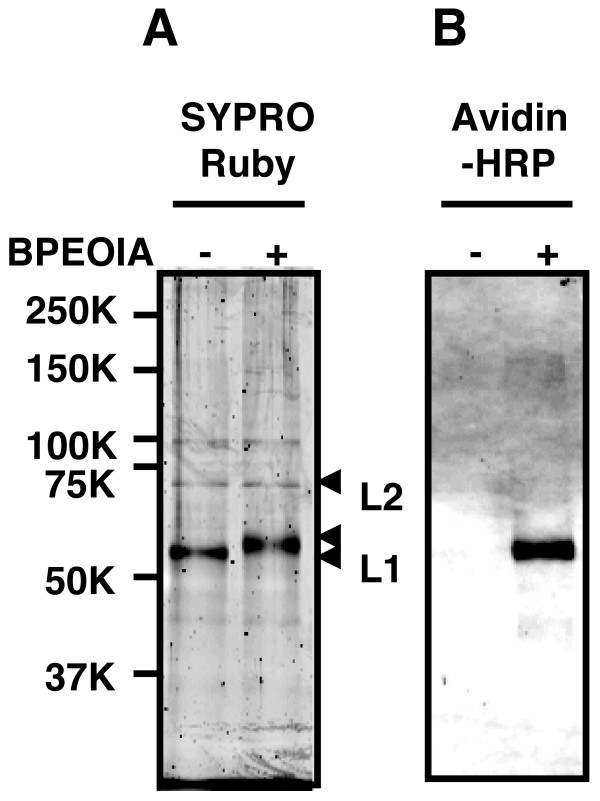
**Binding of BPEOIA to L1 of the 16PV**. (A) The 16PVs were incubated with 1 mM biotin-PEO-iodoacetamide (1 mM) in DMEM at 37°C for 2 h, electrophoresed on an SDS-polyacrylamide gel, and stained with SYPRO Ruby. (B) The proteins in the gel were transferred to a polyvinylidene difluoride membrane and probed with Streptavidin-HRP.

BPEOIA, capable of making a complex with streptavidin, was found to bind to the free thiol of the cysteine residues of L1 of 16PV, which was produced by packaging of a reporter plasmid into an HPV16 capsid. Purified 16PVs were incubated with 1 mM BPEOIA at 37°C for 2 h. The resultant 16PVs were electrophoresed on an SDS-polyacrylamid gel and the separated proteins were stained by SYPRO Ruby (Fig. 3A) or transferred to a membrane. The membrane was probed by horseradish peroxidase (HRP) conjugated streptavidin (Fig. 3B). After the incubation of 16PVs with BPEOIA, the molecular mass of L1 shifted from 55 kDa to 57 kDa (Fig. 3A). The molecular mass of L2 (68 kDa) was not affected by the incubation. The streptavidin made a complex with only 57 kDa L1 (Fig. 3B). The data indicate that BPEOIA bound to the free thiol of cysteine residue(s) of L1.

**Figure 4:**
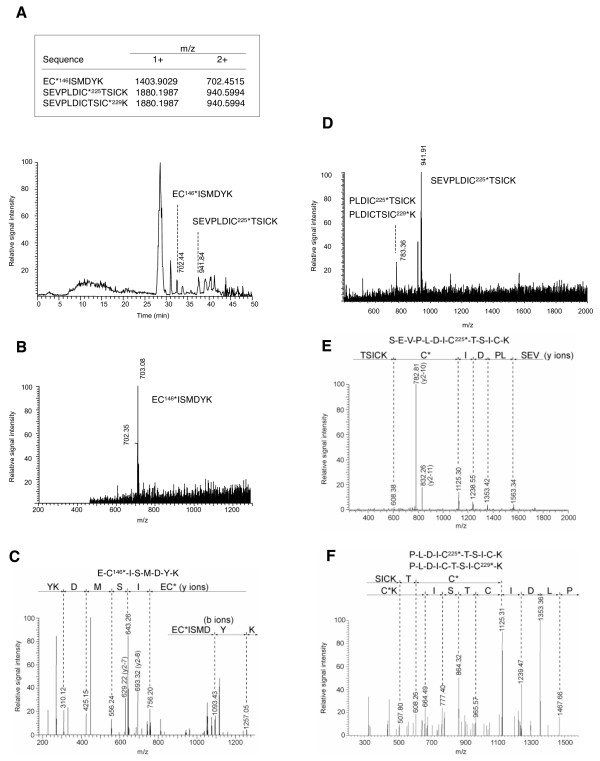
**Analysis of L1-cysteine residues that have bound to BPEOIA by mass spectrometry**. (A) Chromatogram of the tryptic peptides of L1 that bound to BPEOIA. The upper box shows calculated monoisotopic mass value of mono (1+) and doubly (2+) charged masses of three peptides. The asterisk denotes a cysteine bound with BPEOIA. (B and D) Full scan mass spectra corresponding to the EC^146^*ISMDYK (B) and SEVPLDIC^225^*TSICK (D). (C, E and F) MS/MS spectra of corresponding to EC^146^*ISMDYK (C), SEVPLDIC^225^*TSICK (E), PLDIC^225^*TSICK and PLDICTSI C^229^*K (F).

The 57 kDa L1/BPEOIA complex was digested with trypsin and the fragments complexing with BPEOIA were selectively obtained by column chromatography with the streptavidin-resin. The molecular mass of the fragments was measured by liquid chromatography-electrospray ionization-tandem mass spectrometry (LC-ESI-MS/MS) (Fig. 4). The mass of the three fragments, ECISMDYK, SEVPLDICTSICK, and SEVPLDICTSICK, matched with the calculated mass, indicating that BPEOIA bound to the thiol of C146, C225, and C229. Some of the large tryptic fragments that bound to BPEOIA may not be detected because of their low recovery from LC and/or inefficiency in the ionization.

### Sedimentation and morphology of the 16PVs that have bound to DTNB or NEM

**Figure 5:**
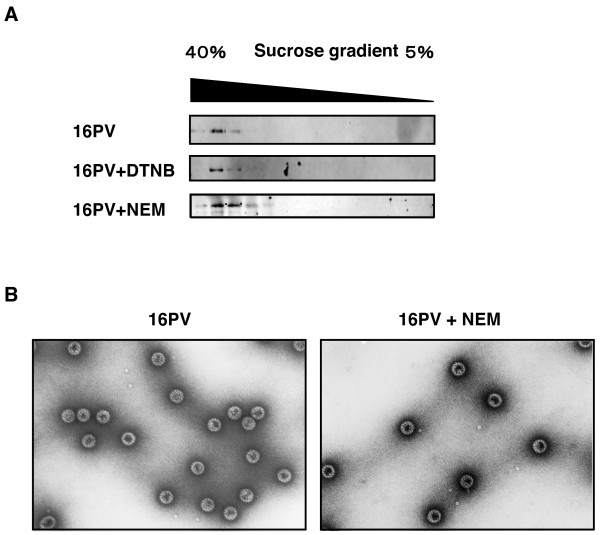
**Sedimentation and morphology of the 16PVs that have bound to DTNB or NEM**. (A) The 16PVs were incubated with DTNB (2 mM) or NEM (2 mM) at 37°C for 2 h. The sample was loaded on the top of a linear sucrose-density gradient (5 to 40%) and centrifuged. L1 in the fractions obtained by a bottom puncture was detected by immunoblotting with mouse anti-HPV16L1 antibody. (B) The 16PVs were incubated with NEM (2 mM) at 37°C for 2 h and observed under a transmission electron microscope.

The 16PVs that had been incubated with DTNB (2 mM) or NEM (2 mM) sedimented through the sucrose gradient (5–40%) as the normal 16PVs did (Fig. 5A). The 16PVs pre-incubated with MBTA or MTSET sedimented similarly (data not presented). These results strongly suggest that the 16PVs that had bound to these reagents were morphologically similar to the normal 16PVs and did not make aggregates.

The 16PVs that had bound to NEM (NEM-16PVs) were not distinguishable from the normal 16PV by an electron microscopy (Fig. 5B). The 16PVs that had been incubated with NEM (2 mM) at 37°C for 2 h were negatively stained with 4% uranylacetate and examined under a transmission electron microscope. Any morphological abnormalities of the NEM-16PVs were not detectable at a magnification of 1:200,000.

### Binding to HeLa cells, internalization, and trafficking of the 16PVs that have bound to DTNB or NEM

**Figure 6:**
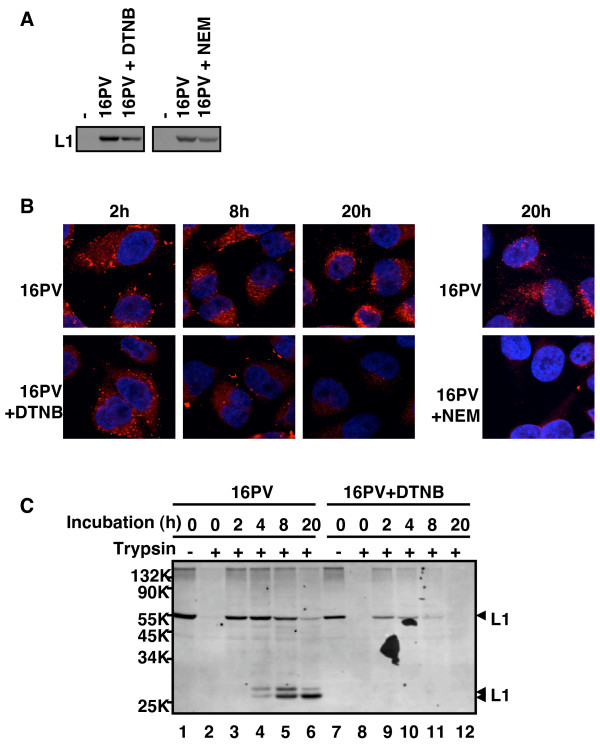
**Binding, trafficking, and degradation of the 16PVs that have bound to DTNB or NEM**. (A) The 16PVs were incubated with DTNB(2 mM) or NEM (2 mM) at 37°C for 2 h and added to HeLa cells. After incubation at 4°C for 1 h, the cells were washed by PBS and lysed. The lysate was electrophoresed on an SDS-polyacrylamide gel. L1 was detected by immunoblotting with anti-HPV16L1 antibody. (B) The 16PVs incubated with DTNB or NEM were added to HeLa cells and incubated for 1 h at 4°C. The cells were cultured at 37°C for 2, 4, 8 or 20 h and fixed. L1 was detected by rabbit anti-HPV16L1 antibody and goat anti-rabbit IgG conjugated with Alexa Fluor 546 (red). DNA was stained with DAPI (blue). (C) The 16PVs incubated with DTNB were added to HeLa cells and incubated for 1 h at 4°C. The cells were harvested with PBS containing 2.5 mM EDTA (for trypsin – sample at 0 h) or with trypsin (for trypsin + sample at 0 h). The rest of cells were cultured at 37°C for 2, 4, 8 or 20 h and harvested with trypsin. The cells were lysed and the lysates were electrophoresed on an SDS-polyacrylamide gel. L1 was detected by immunoblotting with anti-HPV16L1 antibody.

The DTNB-16PVs and the NEM-16PVs were found to bind to HeLa cells less efficiently than the normal 16PVs did. The 16Pvs that had been incubated with DTNB (2 mM) or NEM (2 mM) were inoculated to HeLa cells with incubation at 4°C for 1 h. After a wash with cold PBS to remove the unbound 16Pvs, the cells were lysed immediately. Proteins in the lysate were separated by SDS-polyacrylamid gel electrophoresis (PAGE) and transferred to a membrane. L1 on the membrane was detected with mouse anti-HPV16L1 antibody and goat anti-mouse IgG-HRP (Fig. 6A). The levels of L1 from the DTNB-16PVs and the NEM-16PVs were 50–60 % of that of L1 from the normal 16 PVs, indicating that DTNB and NEM reduced the cell-binding ability of the 16PVs. But the reduction of the binding efficiency does not fully account for the inhibition of the infectivity of the DTNB-16PVs and the NEM-16PVs.

We found that the DTNB-16PVs and the NEM-16PVs disappeared on their way to the nucleus (Fig. 6B). The 16PVs incubated with DTNB (2 mM) or NEM (2 mM) were inoculated to HeLa cells with incubation at 4°C for 1 h. After a wash with the growth medium the cells were incubated further with the growth medium for 2, 8 or 20 h, fixed with paraformaldehyde (4 %), and permeated by Triton-X100. L1 was stained with rabbit anti-HPV16-L1 antibody [12] and anti-rabbit-IgG goat antibody conjugated with Alexa Fluor 546. Nuclear DNA was stained with DAPI. The localization of L1 and DNA was observed under a confocal microscope (Fig. 6B). Although the normal 16PVs reached the perinuclear region and accumulated there at 20 h, the DTNB-16PVs and the NEM-16PVs became undetectable at 20 h.

Consistent with the above observation by confocal microscopy, the immunoblotting to detect L1 showed that the DTNB-16PVs were rapidly degraded in the cells (Fig. 6C). HeLa cells were inoculated with the 16PVs pre-incubated with DTNB (2 mM), and the infected cells were incubated at 4°C for 1 h. After a wash with cold PBS the cells were incubated with trypsin to digest the 16PVs that had not entered the cells. Then, the cells were lysed and subjected to PAGE. The intracellular L1 levels were analysed by immunoblotting with mouse anti-HPV16L1 antibody. The cell-bound 16PVs (lanes 1 and 7) were sensitive to the trypsin digestion (lanes 2 and 8) and became resistant after the incubation at 37°C for 2 h (lanes 3 and 9). The cells inoculated with the 16PVs were further incubated with the growth medium at 37°C for 2, 4, 8, and 20 h, digested with trypsin, and analysed. Fig. 6C clearly showed that the DTNB-16PVs were degraded rapidly and became almost undetectable at 8 h (lane 11).

Thus, the DTNB-16PVs had reduced capability of binding to the cells and enhanced sensitivity to the cellular mechanisms controlling the degradation of foreign proteins.

### Infectivity of the 16PVs composed of mutant L1s having a replacement of cysteine with alanine

A mutational analysis was unsuccessful to identify one particular cysteine residue having the free thiol essential for the infectivity of the 16PV. We newly constructed the L1 mutants by replacement of the cysteine, except for C175 and C428, with alanine. C161A (C161 was replaced with A) was extremely unstable in 293TT cells and was not available for the analysis. The yields of C157A, C229A, C342A, and C379A were very low. Therefore, the number of HeLa cells infected with the mutant PVs was normalized to the content of L1 (Table 1). The infectivity of the mutant PVs, including C146A, C225A, and C229A, ranged between 58 and 187 % of the infectivity of the normal 16PV.

**Table 1:** Infectiviy of mutant 16PVs

	**L1 in PV stock**		**Infectious units**		
		
	**ng of L1/μl**	**%**	**units/μg of L1**	**%**	**SD**
WT	82.0	100	1.77.E+05	100	17.5
C102A	29.3	35.7	1.05.E+05	59.3	1.40
C146A	54.9	66.9	1.64.E+05	92.4	3.37
C157A	3.46	4.22	8.60.E+04	48.1	2.04
C161A	ND	-	NT	-	-
C185A	33.8	41.2	1.62.E+05	91.4	3.86
C225A	81.2	99.0	2.82.E+05	159	4.46
C229A	8.53	10.4	9.60.E+04	54.6	5.59
C324A	5.85	7.14	8.50.E+04	50.2	2.47
C345A	44.3	54.0	3.30.E+05	187	4.03
C379A	13.1	16.0	1.62.E+05	91.4	4.43

ND: not detected NT: not tested

## Discussion

In this study we found that the thiol-reactive reagents bound to C146, C225, and C229 of the 16PVs. In the 3-dimentional-structure model of L1 [15], C146 is involved in forming the DE-loop and C225 and C229 are involved in forming the EF-loop. Because the loops are generally flexible and because C146 and C225 are located at the surface region of the loops and C229 is located near the surface region, it is likely that thiol-reactive reagents easily access the thiols of these cysteine residues. Although it is difficult to examine experimentally whether all or part of the cysteine residues have free thiols, we presume the great majority of C146, C225, and C229 may have free thiols, because the incubation of the 16PVs with the thiol-reactive reagents induced a large effect on their infectivity. The 16PVs lost their infectivity after binding of the thiol-reactive reagents to the free thiols. The DTNB-16PVs and the NEM-16PVs, whose C146, C225, and C229 carried DTNB and NEM as additional side chains, respectively, bound to HeLa cells less efficiently and were degraded rapidly in the cells. Although the 16PV mutants with two or three Ala substitutions for C146, C225, or C229 were too unstable to be used in the infectivity analysis, we obtained the three mutants with an Ala substitution (C146A, C225A, and C229A) and found that the substitution did not affect the infectivity much. Therefore, it is likely that the steric bulk of DTNB or NEM occludes a neighboring portion of the virion involved in the entry and trafficking processes. But there remains a possibility that the disulfide bonding between an unidentified cellular protein(s) and the two remaining cysteine residues in the mutants plays an additive role in the viral entry and trafficking.

It has been reported that cell-surface protein disulfide isomerase (PDI) is required for the entry process of several viruses including mouse polyomavirus. The siRNA-mediated down regulation of PDI of HeLa cells prevents the cells from being infected with mouse polyomavirus [16]. Inactivation of the cell-surface PDI by adding the thiol-reactive reagents, such as DTNB, which do not permeate the membrane, to the culture medium results in the inhibition of entry of HIV1 and Newcastle disease virus [17,18], suggesting that the modified envelope conformation induced by reforming the disulfide-bonding is required for the membrane fusion, which is the essential step for the virus entry [18,19]. However, pre-incubation of the cells with DTNB did not inhibit infection with the 16PV, indicating that modification of the disulfide-bonding in the capsid by cell-surface PDI is not involved in the early steps of HPV infection. The data are consistent with the recent report that reducing agents, such as DTT and 2-ME, do not inhibit HPV infection [20].

Because the thiol-reactive reagents tested in this study bound to the free thiol of the 16PVs at a concentration not toxic for HeLa cells, these reagents might function as practical inhibitors of HPV infection. It would be necessary to test the efficacy and safety of the reagents in animal models.

## Conclusion

HPV16 L1 C146, C225, and C229 have free thiol, which is accessible by the thiol-reactive reagents, such as BPEOIA, DTNB, and NEM. The HPV16 pseudovirions carrying these thiol-reactive reagents lost infectivity by mainly the rapid degradation in the cytoplasm.

## Methods

### Cells

293TT cells, a human cell line expressing a high level of SV40 T antigen, was a kind gift from J. T. Schiller (National Cancer Institute, USA). The cells were cultured in Dulbecco's modified minimal essential medium (DMEM) (No. 21063, Invitrogen Corp., Carlsbad, CA) supplemented with 10% heat-inactivated fetal bovine serum (FBS), 1% non-essential amino acids (Invitrogen Corp.), 1% GlutaMax-I (Invitrogen Corp.), penicillin G potassium (100 units/ml) (Meiji seika Ltd., Tokyo, Japan), kanamycin sulfate (60 μg/ml) (Wako pure chemical industries Ltd. Tokyo, Japan) (growth medium) and hygromycine B (400 μg/ml) (Invitrogen Corp.) in 5% CO2 at 37°C. HeLa cells and SiHa cells were cultured in DMEM supplemented 10% FBS, penicillin G potassium, and kanamycin sulfate.

### Plasmids

pYSEAP, ph16L1, and ph16L2 were gift from J. T. Schiller. pEF1a-EGFP was newly constructed by an insertion of EGFP gene derived from pCMS-EGFP (Clonthech laboratories Inc., Mountain View, CA) into the backbone of pYSEAP. Plasmid expressing mutant L1 with a substitution of alanine for cysteine was constructed by overlap extension PCR method [21] using KOD plus polymerase (TOYOBO Corp., Osaka, Japan) and ph16L1 as template. 5'-GCTGGTGTGGGCCGCCGTGGGCGTGGAG-3' and 5'-CCTCCACGCCCACGGCGGCCCACACCAGCC-3' were used as forward (F) and reverse (R) primers to replace C102 with A, respectively. Following oligonucleotides were used as primers to introduce the other mutantions: 5'-CGACAACAGGGAGGCCATCAGCATGGACTACAAG-3' (F for C146A), 5'-GTAGTCCATGCTGATGGCCTCCCTGTTGTCCAC-3' (R for C146A), 5'-CAAGCAGACCCAGCTGGCCCTGATCGGCTGCAAG-3' (F for C157A), 5'-CTTGCAGCCGATCAGGGCCAGCTGGGTCTGCTTG-3' (R for C157A), 5'-CTGTGCCTGATCGGCGCCAAGCCCCCCATCG-3' (F for C161A), 5'-CGATGGGGGGCTTGGCGCCGATCAGGCACAG-3' (R for C161A), 5'-AACCCCGGCGACGCCCCCCCCCTGGAGCTG-3' (F for C185A), 5'-CAGCTCCAGGGGGGGGGCGTCGCCGGGGTTC-3' (R for C185A), 5'-GTGCCCCTGGACATCGCCACCAGCATCTGCAAG-3' (F for C225A), 5'-CTTGCAGATGCTGGTGGCGATGTCCAGGGGCAC-3' (R for C225A), 5'-ACCAGCATCGCCAAGTACCCCGACTACATC-3' (F for C229A), 5'-ATGTAGTCGGGGTACTTGGCGATGCTGGTGCAGATG-3' (R for C229A), 5'-CAACAACGGCATCGCCTGGGGCAACCAGCTGTTC-3' (F for C324A), 5'-GAACAGCTGGTTGCCCCAGGCGATGCCGTTGTTGTG-3' (R for C324A), 5'-CCAACATGAGCCTGGCCGCCGCCATCAGCAC-3' (F for C345A), 5'-GTGCTGATGGCGGCGGCCAGGCTCATGTTGGTGCTCC-3' (R for C345A), 5'-CATCTTCCAGCTGGCCAAGATCACCCTGAC-3' (F for C379A), 5'-GTCAGGGTGATCTTGGCCAGCTGGAAGATGAACTG-3' (R for C379A). 5'-TGCCTTTACTTCTAGGCCTGTACG-3' and 5'-TGCTCCTGGTGGTGTCCACCACGGTC-3' were used as primers to join the part containing the mutation back to the rest of the entire L1 gene of C146A, C157A, C161A, C185A, C225A, C229A. 5'-AACCTGGCCAGCAGCAACTACTTCCC-3' and 5'-AACTAGAAGGCACAGTCGAGGCTG-3' were used similarly to produce C324A, C345A, C379A. The resultant DNA fragments were inserted into ph16L1 after digestion with NotI and ApaI (for C146A, C157A, C161A, C185A, C225A, and C229A) or with ApaI and HindIII (for C324A, C345A, and C379A).

### Thiol-reactive reagents

Biotin polyethyleneoxide iodoacetamide (BPEOIA) was purchased from SIGMA-ALDRICH Corp. (Saint Luis, MO). N-ethylmaleimide (NEM) was purchased from Nakarai Tesque. Inc. (Kyoto, Japan). 5,5'-dithiobis(2-nitrobenzoic acid) (DTNB) was purchased from SIGMA-ALDRICH Corp (Saint Luis, MO). 4-(N-maleimido)benzyl-trimethylammonium iodide (MBTA), and [2-(trimethylammonium)ethyl] methanethiosulfonate bromide (MTSET) were purchased from Toronto Research Chemicals Inc. (Toronto, Canada)

### Preparation of 16PV

The 16PV, an HPV16 capsid containing a reporter plasmid expressing EGFP, was produced by the previously described procedure [6,7,12] with minor modification. 293TT cells (40% confluent in 10-cm culture dish) were transfected with mixture of ph16L1 (13.5 μg), ph16L2 (3 μg), and pEF1a-EGFP (13.5 μg) by using Optifect (Invitrogen Corp.) in OPTI-MEMII (Invitrogen Corp.). After incubation for 3 days, the cells were scraped off and suspended in 0.5 ml lysis buffer (PBS containing 9.5 mM MgCl_2_, 0.35% Brij 58, [Sigma-Aldrich Inc., St. Louis, MO], 0.1% Benzonase [Sigma-Aldrich Inc.], 0.1% Plasmid Safe ATP dependent-DNase [EPICENTRE Corp. Madison, WI], 1 mM ATP) and incubated at 37°C for 20–24 h with slow rotation. The lysate was cooled on ice for 5 min, mixed with 1/4 volume of 5 M NaCl solution, and kept on ice for 10 min, then, centrifuged at 5,000 × g at 4°C for 10 min. The resultant supernatant was laid on an Optiprep gradient composed of 27%, 33%, and 39% in PBS containing 1 mM CaCl_2_, 0.5 mM MgCl_2_, 2.1 mM KCl, and 0.8 M NaCl and centrifuged at 47,900 rpm at 16°C for 3 h with SW50.1 rotor (Beckman Coulter Inc. Fullerton, CA). The fraction containing the purified 16PVs was collected by puncturing the bottom and used as the stock.

### Infectivity assay

The 16PV stock was diluted at 10-fold with DMEM and received BPEOIA (1 mM); the 16PV stock was diluted at 10-fold with the growth medium and received DTNB (2 mM), NEM (2 mM), MBTA (2 mM), or MTSET (2 mM). The mixtures were incubated at 37°C for 2 h and diluted with the growth medium at 1,000-fold. HeLa cells (1.5 × 10^5^) in a well of a 24-well culture-plate were inoculated with the sample and cultured for 2 days. The cells were harvested with trypsin. EGFP-positive cells were counted by a fluorescence activated cell sorting (FACS Calibar, Becton Dickinson and Company Ltd., San Joe, CA).

### Binding of BPEOIA to 16PVs

BPEOIA was dissolved in H_2_O to 18.4 mM. The 16PV stock was diluted at 10-fold with DMEM (No. 21063, Invitrogen Corp.) containing BPEOIA (1 mM) and incubated at 37°C for 2 h. Then, DTT (100 mM), which reacted with remaining excess BPEOIA, was added to the mixture and incubated at 37°C for 30 m (BPEOIA+ sample). The 16PV stock was diluted at 10-fold with DMEM, incubated at 37°C for 2 h, mixed with DTT (100 mM), and further incubated at 37°C for 30 m. Then, BPEOIA (1 mM) was added to the mixture and incubated at 37°C for 30 m (BPEOIA- sample). The 16PVs were concentrated by using a PAGEprep Advance Kit (PIERCE Biotechnology Inc., Rockford, IL) and suspended in the SDS sample buffer (50 mM Tris-HCl pH 6.8, 5% glycerol, 2 % SDS, and bromphenol blue) containing 100 mM DTT. The sample was boiled and electrophorased on an SDS-polyacrylamide gel. The proteins in the gel were stained with SYPRO Ruby (Invitrogen Corp.) or transferred to membrane Hybond-P (GE Healthcare Bio-Science AB, Uppsala, Sweden). The membrane was blocked with skim milk and incubated with the horseradish peroxidase (HRP) conjugated-streptavidin (GE Healthcare Bio-Science AB). The HRP activity was detected by using an ECL plus western blotting detection system (GE Healthcare Bio-Science AB) and Typhoon 9410 (GE Healthcare Bio-Science AB).

### Analysis by liquid chromatography electrospray ionization-tandem mass spectrometry (LC-ESI-MS/MS)

The HPV16 pseudovirions that bound to BPEOIA were separated by SDS-PAGE and stained by SYPRO Ruby as described above. The gel pieces containing the L1/BPEOIA complex were excised and the L1 in the gel was digested with trypsin (Trypsin Gold Mass Spectrometry Grade, Promega Corp., Madison WI) as previously described [22]. The digested peptides were extracted from the gel pieces by one change of NH_4_HCO_3 _(20 mM) and 4 changes of acetonitrile (50 %). The peptides were suspended in PBS by adding equal volume of 2× concentrated PBS and then incubated with monomeric avidin beads (Ultralink Immobilized Monomeric Avidin, PIERCE Biotechnolog Inc.). The beads were washed with PBS twice, with methanol (20 %) in NH_4_HCO_3 _(50 mM) twice, and with water twice. The peptides that bound to the beads were eluted with acetonitrile (30 %) containing TFA (0.4 %). After volatilization of acetonitrile the peptides were analyzed by a liquid chromatography (MAGIC 2002 system, Michrome Bioresources Inc., Auburn, CA) equipped with C18 column (Inertsil EX-Nano ODS-3, 0.1 mm i.d. × 50 mmL, GL Sciences Inc, Tokyo, Japan) coupled with a nano spray apparatus (AMR Inc. Tokyo, Japan) for electrospray ionization-iontrap mass spectrometry (LC-ESI-IT-MS) (LCQ-decaXP, Thermo electron corp., San Jose, CA). The data were collected by data-dependent mode and the MS/MS sequence were analysed by using software Bioworks (Ver.3.1, Thermo electron corp.) with variable modification option of a mass unit of 414.19 for the biotin polyethleneoxide moiety.

### Sedimentation assay

The 16PV stock was diluted at 10-fold with the growth medium, and DTNB (2 mM) was added to the medium. The 16PVs were then incubated for 2 h at 37°C. The sample was loaded on a linear sucrose-density gradient (5 to 40%) in PBS. After centrifugation at 120,000 × g at 4°C for 2.5 h with and SW50.1 rotor, aliquots (400 μl) were collected. Ten μl of the aliquot was mixed with an equal volume of the 2× concentrated SDS-sample buffer containing 100 mM DTT, boiled, and electrophorased on an SDS-polyacrylamide gel. The proteins were transferred to a Hybond-P membrane (GE Healthcare Bio-Science AB). The membrane was blocked with skim milk, incubated with mouse anti-HPV type 16L1 antibody (BD Biosciences Pharmingen Com., San Diego, CA), and then incubated with anti-mouse IgG-HRP (Santa Cruz Biotechnology Inc., Santa Cruz, CA). The HRP activity was detected by using an ECL plus western blotting detection system (GE Healthcare Bio-Science AB) and Typhoon 9410 (GE Healthcare Bio-Science AB).

### Electron microscopy

The 16PV stock was mixed with NEM (2 mM) and incubated at 37°C for 2 h. The excess NEM was removed by using a Bio-Spin 30 column (Bio-Rad Laboratories Inc., Hercules, CA) equilibrated with phosphate buffer containing 0.5 M NaCl. The 16PVs were concentrated with a Microcon YM-100 (Millipore Corp., Bedford, MA) and then settled on carbon-coated copper grids. The 16PVs were negatively stained with 4% uranylacetate and examined in a transmission electron microscope (Hitachi model H-7650, Hitachi corp., Tokyo, Japan).

### Binding assay

The 16PV stock was diluted at 20-fold with the growth medium, and DTNB (2 mM) was added to the medium. The stock was incubated at 37°C for 2 h and used for the binding assay. The 16PV stock was mixed with NEM (2 mM) and incubated at 37°C for 2 h. Then, the excess NEM was removed by using a Bio-Spin 30 column as described above was used for the binding assay. These samples were diluted with the growth medium at 20-fold and added to HeLa cells (1.5 × 10^5^). The cells were incubated at 4°C for 1 h, washed with PBS, harvested with PBS containing 2.5 mM EDTA, and lysed. The lysate was electrophorased on an SDS-polyacrylamide gel. The separated proteins were transferred to a polyvinylidene difluoride membrane and L1 was detected by immunoblotting with mouse anti-HPV16 L1 anitbody and anti-mouse IgG-HRP.

### Immunofluoresence microscopy

HeLa cells (1.5 × 10^5^) were seeded onto a well of a 4-chamber glass slide (BD Biosciences Falcon, Bedford, MA) with the growth medium. The cells were inoculated with the 16PV samples similarly prepared as the samples for the biding assay and incubated at 4°C for 1 h. The cells were washed with the growth medium and incubated at 37°C for 2, 8 or 20 h. The cells were fixed with PBS containing paraformaldehyde (4%) at room temperature (RT) for 10 m and washed with PBS. The cells were made permeable with PBS containing Triton X-100 (1%) at RT for 10 m and washed with PBS. The cells were incubated with rabbit anti-HPV16 L1 serum [12] in PBS containing BSA (3%) at RT for 1 h, washed with PBS containing Tween-20 (0.2%), incubated with Alexa Fluor 546 goat anti-rabbit IgG (H+L) (Invitrogen Corp.) in PBS containing BSA (3%), and washed with PBS containing Tween-20 (0.2%). The cells were coated with a ProLong Gold anti-fade reagent with DAPI (Invitrogen Corp.) and imaged in a FLUOVIEW FV1000 confocal microscope (OLYMPUS, Tokyo, Japan).

### Internalizaiton assay

HeLa cells (1.5 × 10^5^) in a well of a 24-well culture plate were inoculated with the 16PVs that had been preincubated with DTNB as done for the binding assay. The cells were incubated at 4°C for 1 h. The cells for the samples at 0 h were washed with the growth medium and immediately harvested with PBS containing 2.5 mM EDTA or with PBS containing trypsin. The other cells were incubated with the growth medium at 37°C for 2, 4, 8 and 20 h and harvested with PBS containing trypsin. The cells were lysed and electrophorased on an SDS-polyacrylamide gel. The separated proteins were transferred to a polyvinylidene difluoride membrane. L1 was detected by immunoblotting with mouse anti-HPV16 L1 anitbody and anti-mouse IgG-HRP.

## Competing interests

The author(s) declare that they have no competing interests.

## Authors' contributions

YI conceived of the study, carried out the biological experiments, and drafted the manuscript. KK supported the preparation of the 16PV stock. TM constructed the reporter plasmid. KT conducted electron microscopy. FSO and KH conducted the analysis by LC-ESI-IT-MS. TK supervised the study and helped to draft the manuscript.
